# Application of multi-channel *in situ* infrared spectroscopy: the case of PVB thermal aging

**DOI:** 10.1039/d3ra03932c

**Published:** 2023-09-26

**Authors:** Chun Xu, Shuen Liang, Bo Jin, Qian Xiao, Xiaofei Hao, Zhongping Liu, Nannan Lin, Jie Sun, Heliang Sui

**Affiliations:** a State Key Laboratory of Environment-friendly Energy Material, School of Materials Science and Engineering, Southwest University of Science and Technology Mianyang 621010 China; b Institute of Chemical Materials, China Academy of Engineering Physics Mianyang Sichuan 621900 China 15308116490@126.com

## Abstract

Thermal kinetic parameters are important for establishing the relationship between the aging process, time, and temperature, which would help predict the thermal aging lifetime and stability in the application of polymer materials. We developed a multi-channel *in situ* detecting device, which provided an efficient method for IR spectrum measurement. The thermal aging process of polyvinyl butyral (PVB) at several constant temperatures (100 °C, 110 °C, 120 °C, 135 °C, and 150 °C) had been studied by the multi-channel *in situ* infrared reaction device. The kinetic parameters (*E*_α_) were calculated from the absorbance intensity of –C–O–, –C=O, –CH_3_, and –OH. The –OH proved to be the active site of PVB during thermal aging, and a possible thermal aging mechanism of PVB was proposed. We proved the method using a combination of a multi-channel *in situ* reaction device and FTIR was suitable to study the aging mechanism and kinetics of polymers.

## Abstract 2

Thermal kinetic parameters are important for establishing the relationship between the aging process, time, and temperature, which would help predict the thermal aging lifetime and stability in the application of polymer materials.

## Graphical abstract

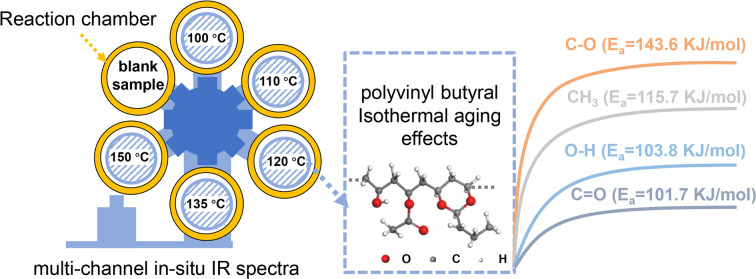

## 1 Introduction

Thermal kinetic parameters are important for the study of a thermal aging lifetime and the stability of polymer materials. In the Arrhenius formula, proposed by S. A. Arrhenius, there are two crucial empirical parameters: the pre-exponential factor (*A*) and *E*_α_. *E*_α_ values can predict a range of chemical reactions, including combustion and explosion, thermal cracking, heterogeneous catalysis, enzyme catalysis, and polymerization. Additionally, *E*_α_ values can provide insight into total package reactions of basic chemical substances, the state of tertiary reactions, and the stability of substances. In the formula's exponential term, any change in *E*_α_ value can significantly affect the chemical reaction rate constant. For instance, a mere 5.7 kJ mol^−1^ alteration in *E*_α_ can increase the reaction rate by tenfold at ambient temperature. Thus, it is crucial to precisely calculate the thermal kinetic parameters' value.

There are a few methods to calculate the dynamic parameters. Such as Kim–Park,^1^ Flynn–Wall–Ozawa,^2,3^ Friedman,^4,5^ Coats–Redfern,^6,7^ Achar,^8,9^ Ozawa,^10,11^ Kissinger–Akahira–Sunose,^12,13^ Kissinger,^14,15^ Freeman–Carroll^16,17^ and Newkirk,^18,19^*etc.* The above kinetic methods are based on the analysis of thermogravimetry (TG) and differential scanning calorimetry (DSC). Usually, the dynamic parameters are obtained from the data of the thermal degradation or thermal structure transformation temperatures that will be higher than the application environment of the polymer materials. High temperatures can cause deviations in the dynamic parameters, rendering the degradation mechanisms ineffective in representing the thermal aging process of polymer materials. Consequently, the accuracy of measurement methods used to calculate dynamic parameters may be called into question when predicting the lifetime of thermally aged materials.^20^ In the isothermal kinetics study of polymers, the dynamic parameters can also be obtained according to the time-dependent changes based on the properties of penetration, softening point, apparent viscosity, elastic strength, *etc.* However, the test of these macroscopic properties would be usually accompanied by large errors or the low sensitivity, which makes it difficult to obtain a better aging regular in the evaluation process. Ultimately, the thermal aging model and the dynamic parameters would not be calculated accurately.

Liau^21,22^ used TG method to calculate the *E*_α_ of PVB in the range of 181–200 kJ mol^−1^. Ivanov^23^ studied the *E*_α_ value of thermal degradation in different temperature ranges from 70–201 kJ mol^−1^. It can be seen that *E*_α_ obtained by thermal degradation method has a large difference. The difference is not conducive to the establishment of the thermal aging model of PVB. In the characterization methods of polymers, *in situ* infrared spectroscopy can directly study the changes of chemical functional groups of condensed matter with time and temperature, which is sensitive to the changes chemical structure.^24^*In situ* FTIR can monitor the change of the absorption peak of the main characteristic groups during the thermal aging process is useful to understand the thermal aging behavior of the polymer. However, when *in situ* FTIR is used to study the isothermal aging effect of polymers, the infrared spectroscopy will be occupied for a long time, which hinders the *in situ* infrared spectroscopy widely used in polymer aging research.

We develop a multi-channel *in situ* novelty device, which provides an efficient method for IR spectrum measurement. The device can simultaneously determine multiple samples at different temperatures, with high efficiency and good repeatability. This device combined with an FTIR spectrometer can obtain the chemical structure evolution at a constant temperature. In this paper, polyvinyl butyral materials were selected to study the thermal aging effects by the multi-channel *in situ* infrared spectroscopy. Aimed to obtain the dynamic parameters and the thermal aging mechanism through the analysis of the *in situ* infrared spectra of the main chemical functional groups of PVB materials during aging and prove the effectiveness of multi-channel *in situ* infrared spectroscopy.

## 2 Experimental

### 2.1 Materials and preparation

**Fig. 1:**
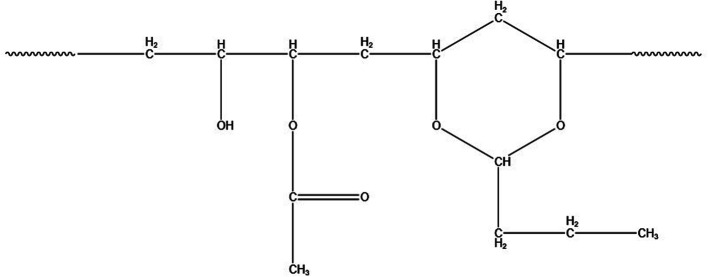
Structure of PVB.

**Fig. 2:**
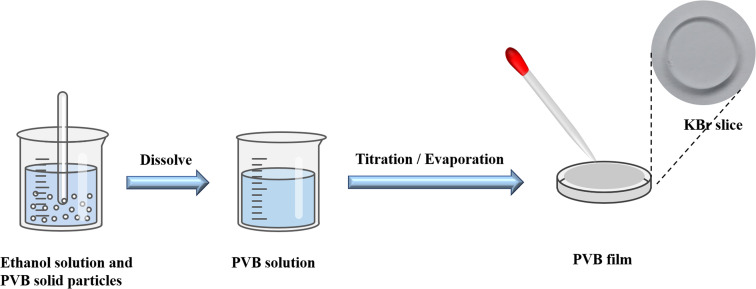
PVB film preparation flowchart.

PVB was supplied by the Institute of Chemical Materials, Chinese Academy of Engineering Physics. The structure of PVB is shown in Fig. 1. KBr salt slices are obtained by rough grinding, fine grinding, and polishing after cutting from the KBr crystal, and the KBr salt slices can be purchased from a company, as shown in Fig. 1. During the polymer sample preparation, we first dissolved the PVB using ethanol. Then the ethanolic solution of PVB was dropped on the KBr salt slices. The sample would be prepared after the ethanol evaporates, as shown in Fig. 2.

### 2.2 Characterization methods

**Fig. 3:**
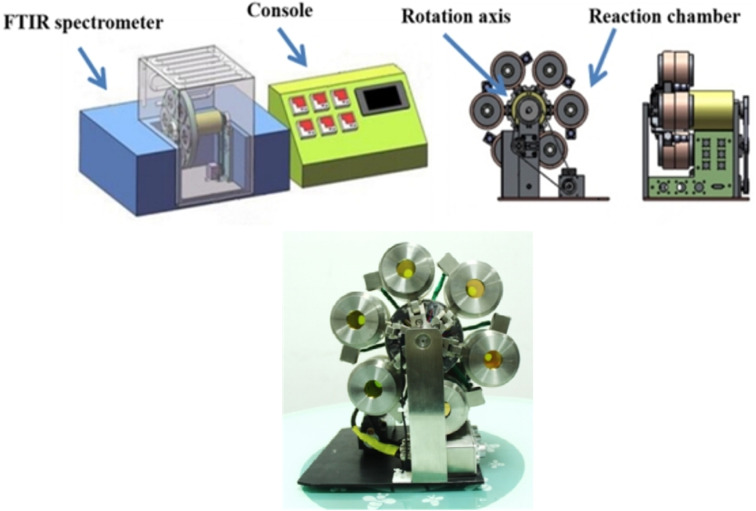
Structure of multi-channel *in situ* reaction system.^25^

The multi-channel *in situ* reaction system (Fig. 3) consists of a console and a reaction system with six reaction chambers. The console desk can transmit instructions to the reaction system, such as temperature, heating rate, single-reaction chamber measurement time and the time for rotating disk to run for one revolution, *etc.* Each reaction chamber of the reaction system is independently controlled by the console desk. *In situ* Fourier transform infrared spectroscopy analysis technique was used to analyze the infrared spectra of PVB under different temperature conditions, instrument: PerkinElmer FTIR Spectrometer Frontier.

The temperatures of six reaction chambers are shown in Table 1. Every reaction chamber can be heated from room temperature to 200 °C, and the six chambers can rotate at a certain time interval that can be set subjectively. In the experiment, the reaction chamber was rotated from the current position to the next position continuing 20 min, and same reaction chamber would be rotated to initial position every two hours. Samples were placed in reaction chambers 1–5 and the blank KBr salt slices were placed in the reaction chamber 6 for background testing. The IR spectra were automatically collected by FT-IR spectrometer every 20 min until 10 d. Spectra were measured with a resolution of 4 cm^−1^ in the range of 4000–600 cm^−1^. The background was automatically corrected each time, and the number of spectra collected for each sample was 122.

**Table 1:** Channel temperatures

Reaction chamber	1	2	3	4	5	6
Temperature/°C	100	110	120	135	150	100
Sample	PVB	PVB	PVB	PVB	PVB	Blank

**Fig. 4:**
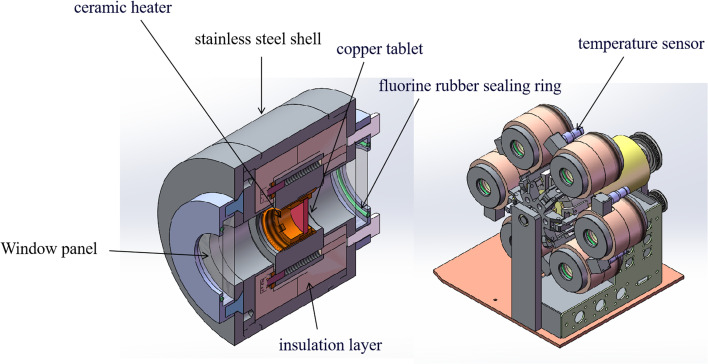
The core heating structure unit of multi-channel *in situ* reaction chamber.

For multi-channel devices, the internal structure of the reaction chamber is shown in Fig. 4. It is mainly composed of the following parts: stainless steel shell, ceramic heater, copper tablet, insulation layer, fluorocarbon rubber sealing and temperature sensor. The role of the ceramic heating element is to generate heat. The copper tablet is used to place the sample. The role of the fluorine rubber sealing ring is to prevent air convection. The insulation layer and the stainless steel shell can maintain the temperature in the reaction chamber and avoid heat loss. In addition, the temperature sensor is connected with the computer program, mainly to monitor the temperature of the reaction chamber and control the ceramic heating body to produce heat, so that the reaction chamber is in a constant temperature state.

**Fig. 5:**
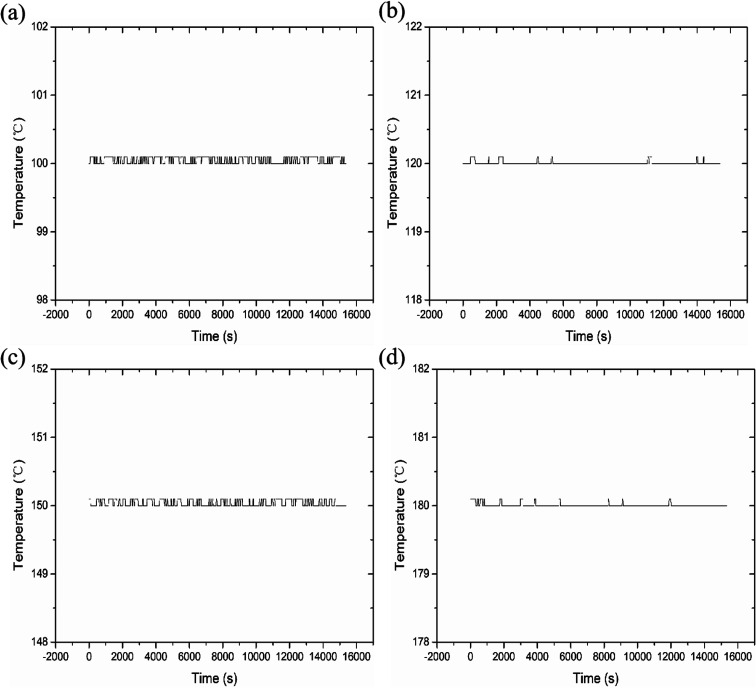
Data at different test temperatures. (a) 100 °C; (b) 120 °C; (c) 150 °C; (d) 180 °C.

We measured four temperatures through different reaction chambers and learned about the temperature state of the reaction chamber through program monitoring, as shown in Fig. 5. The experimental results show that each reaction chamber can independently control the temperature, with an accuracy of ±0.3 °C. It can maintain a constant temperature state, so it is conducive to aging experiments at different temperatures.

And the multi-channel device uses a servo motor to rotate and locate. There are photoelectric sensor marking lines inside the motor, which will be automatically calibrated after each rotation.

The results of the above tests show that the combination of the multi-channel device and the infrared spectrometer can monitor the infrared spectra of multiple sets of samples at different temperatures. It has the characteristics of high efficiency and good repeatability, which can make up for the shortcomings of the current infrared spectroscopy research kinetics.

## 3 Results and discussion

### 3.1 *In situ* FTIR analysis of PVB

**Fig. 6:**
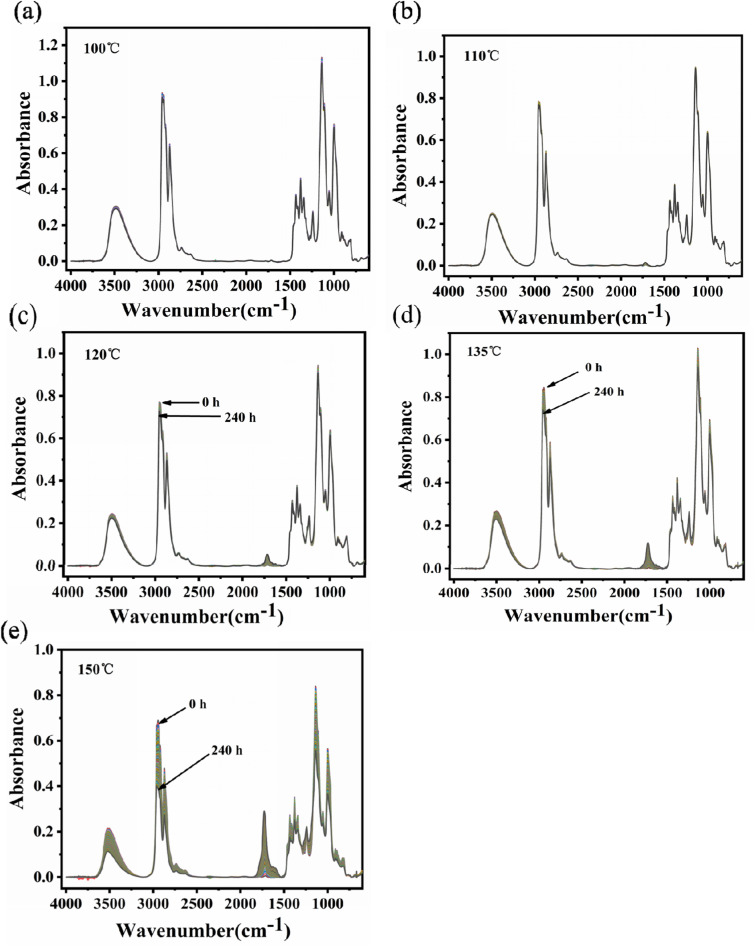
Infrared spectra of PVB at different temperatures: (a) 100 °C, (b) 110 °C, (c) 120 °C, (d) 135 °C, (e) 150 °C.

The infrared spectra of PVB under various aging conditions at 100 °C, 110 °C, 120 °C, 135 °C, and 150 °C were shown in Fig. 6. It was shown that there was no obvious change in the spectra peak shape and intensity of PVB at 110 °C during the aging process, indicating that the molecular structure of PVB changed little below 110 °C. Above 110 °C, the intensity of the characteristic peaks of the spectra decreased or increased with increasing aging time and temperature, indicating that the reaction rate of the chemical structure increasing gradually.

**Fig. 7:**
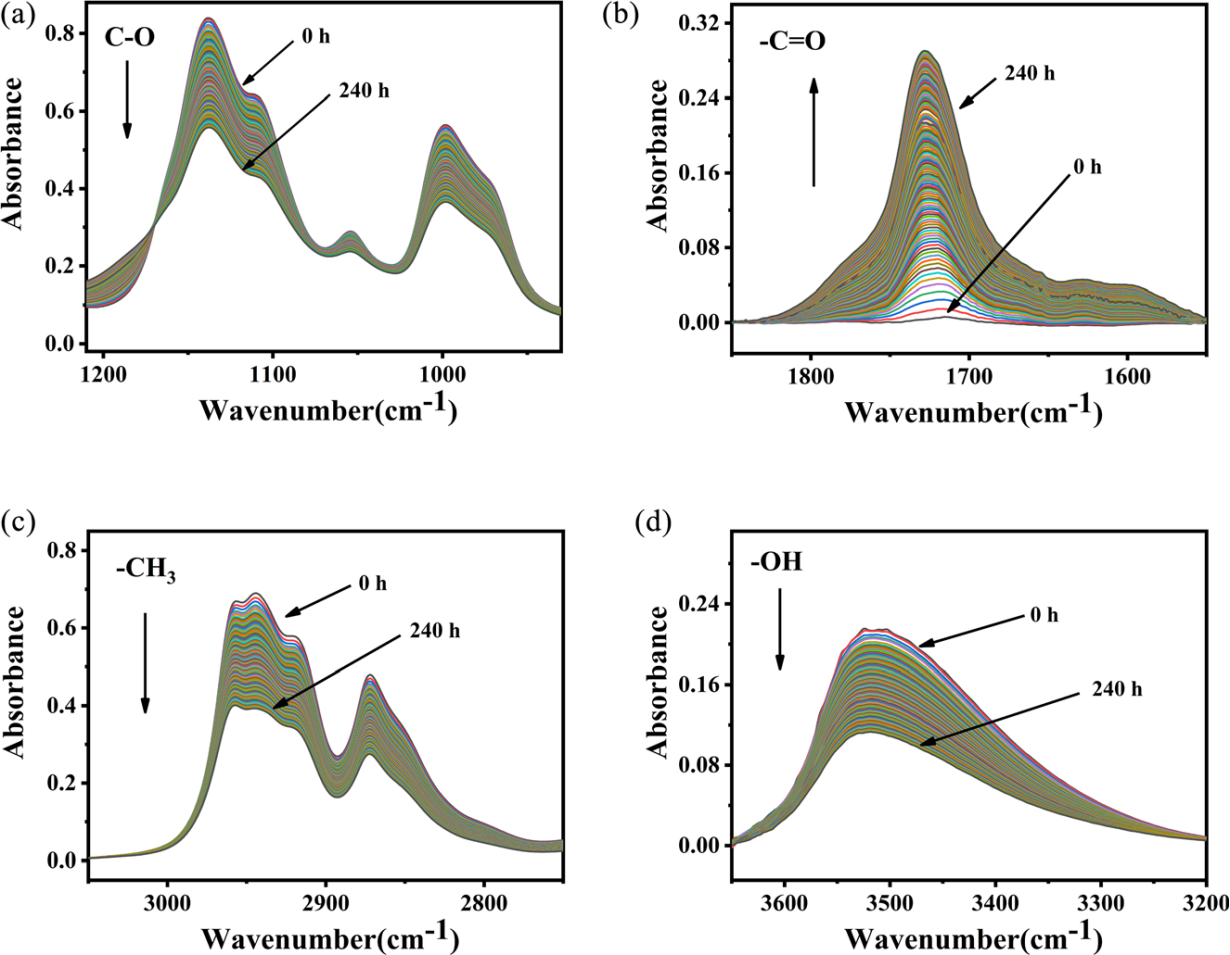
*In situ* FT-IR spectra of PVB at 150 °C. (a) 1210–930 cm^−1^, (b) 1850–1550 cm^−1^, (c) 3100–2750 cm^−1^, (d) 3650–3200 cm^−1^.

In order to analyze the structure evolution of PVB molecular chain clearly, the *in situ* infrared spectra of PVB aged at 150 °C were analyzed in Fig. 7. Infrared absorbance peaks around 3500 cm^−1^ are due to the stretching vibration absorption of hydroxyl groups. The peaks at 2954 cm^−1^, 2943 cm^−1^, and 2920 cm^−1^ are attributed to the stretching vibration absorption of –CH_3_, –CH_2_, and –CH,^26^ respectively. The peaks at 1718 cm^−1^ are the stretching vibration of carbonyl groups. The peaks at 1120 cm^−1^ and 968 cm^−1^ belong to the characteristic peaks of the acetal groups, and the peaks at 1550 to 1650 cm^−1^ represents the double bonds.

It was shown that the absorbance intensity of –OH, –CH_3_ and –C–O– groups decreased with the increase of aging time. Whereas, the absorbance intensity of –C=O groups had the opposite evolution, indicating the formation of aldehydes or ketones during aging. As shown in Fig. 7, temperature had important influence on the changes of the spectra during aging, and the variation become more and more obvious with the increase of aging temperature.

### 3.2 Thermal aging kinetics of PVB

**Fig. 8:**
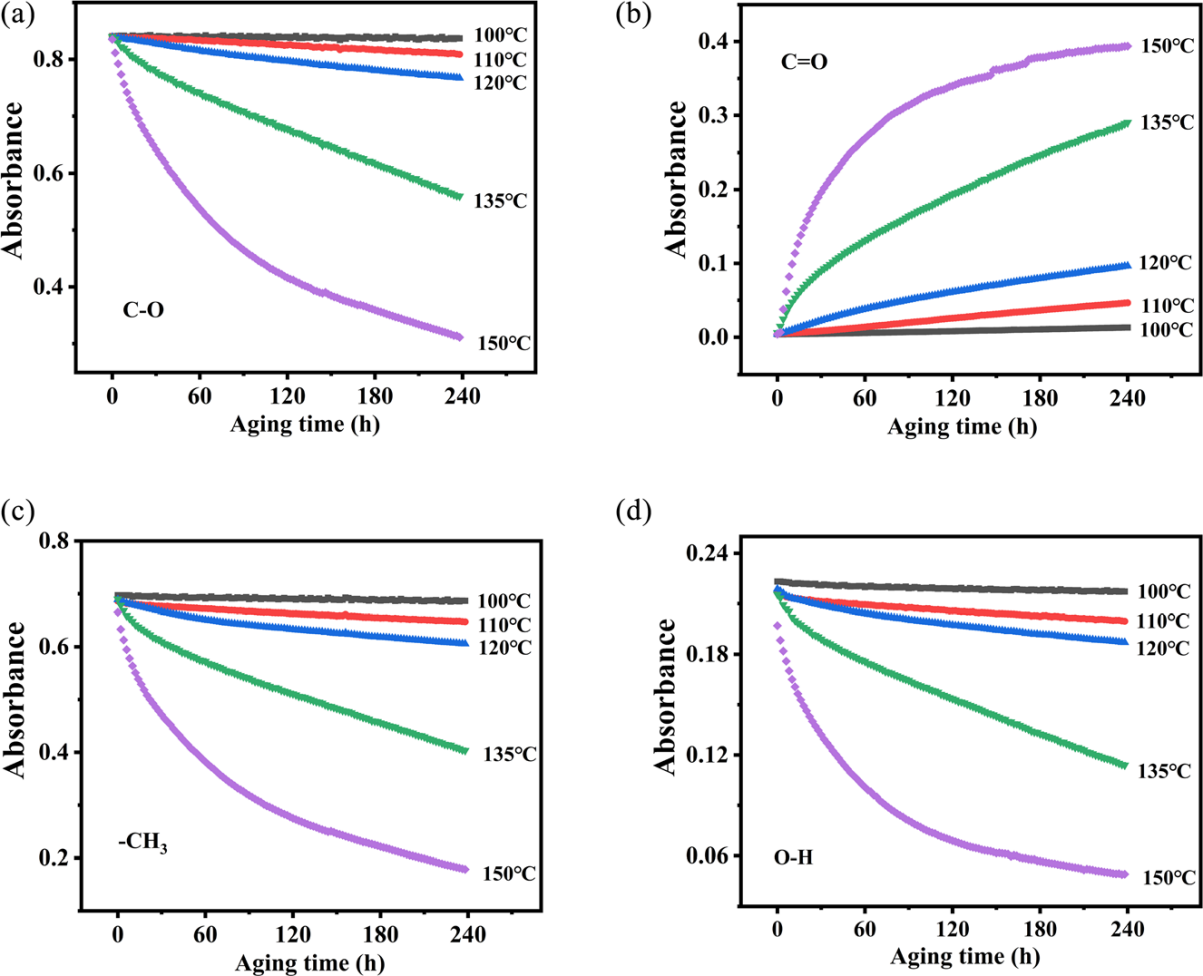
Changes in functional group absorbance and aging time at different temperatures. (a) C–O, (b) C=O, (c) –CH_3_, (d) O–H.

According to the changes in the characteristic peaks in the infrared spectra, we explored the absorbance intensity changes of the four main functional groups of PVB (–CH_3_, –OH, –C=O, and –C–O) at five aging environments (100 °C, 110 °C, 120 °C, 135 °C and 150 °C) shown in Fig. 8. It was shown that the changes of various groups with the aging time increase almost linearly. When the thermal aging temperature was lower than 135 °C, the evolution of the curves was similar. This is different from the absorption intensity changes when the aging temperature was 150 °C. This could be due to the following two reasons. One was that when the temperature exceeded 150 °C, PVB gradually transformed into a viscous fluid state and the molecular mobility of the polymer unit and the rate of chemical processes involving PVB change dramatically, forming a large number of free radicals, thereby accelerating the oxidation of the PVB molecular chain.^26^ The other was that the high temperatures made the aldehydes produced in the degradation process of PVB rapidly oxidize into acids, which play the role of autocatalytic degradation.^27^

Up to now, there are few studies on the kinetics of this process from the perspective of chemical structure. As we all know, the thermal stability of the material is related to its thermal kinetic parameters. The Arrhenius equation and first-order reaction were used to calculate the thermal aging kinetic parameters of PVB.

#### 3.2.1 Thermal kinetic parameters analysis from the zero-order reaction equation

The data of four temperatures that tend to be linear were used to calculate the apparent activation energy of the above four groups by the Arrhenius equation to determine the thermal stability difference between these groups, which is helpful for further exploring the thermal aging mechanism of PVB.

It is assumed that PVB would decompose completely when kept at a constant temperature for an adequate time. The decomposition extent of reaction can be defined as:

1 *α* = (*A*_0_ − *A*_*t*_)/*A*_0_

where *α* is the degree of reaction decomposition, *A*_*t*_ is the real-time absorbance, and *A*_0_ is the initial absorbance.

The empirical formula between temperature and reaction rate is the Arrhenius equation:

2 *k* = *A*e^(−*E*_α_/*RT*)^

where *E*_α_ is the activation energy (kJ mol^−1^), *A* refers to the pre-exponential factor (s^−1^), *R* denotes the gas constant (8.314 kJ K^−1^ mol^−1^), *T* indicates the temperature (K), and *k* represents the reaction rate constant (s^−1^).

Eqn (2) can be converted into logarithmic form, which is the following eqn (3):

3 ln *k* = ln *A* − *E*_α_/*RT*

**Fig. 9:**
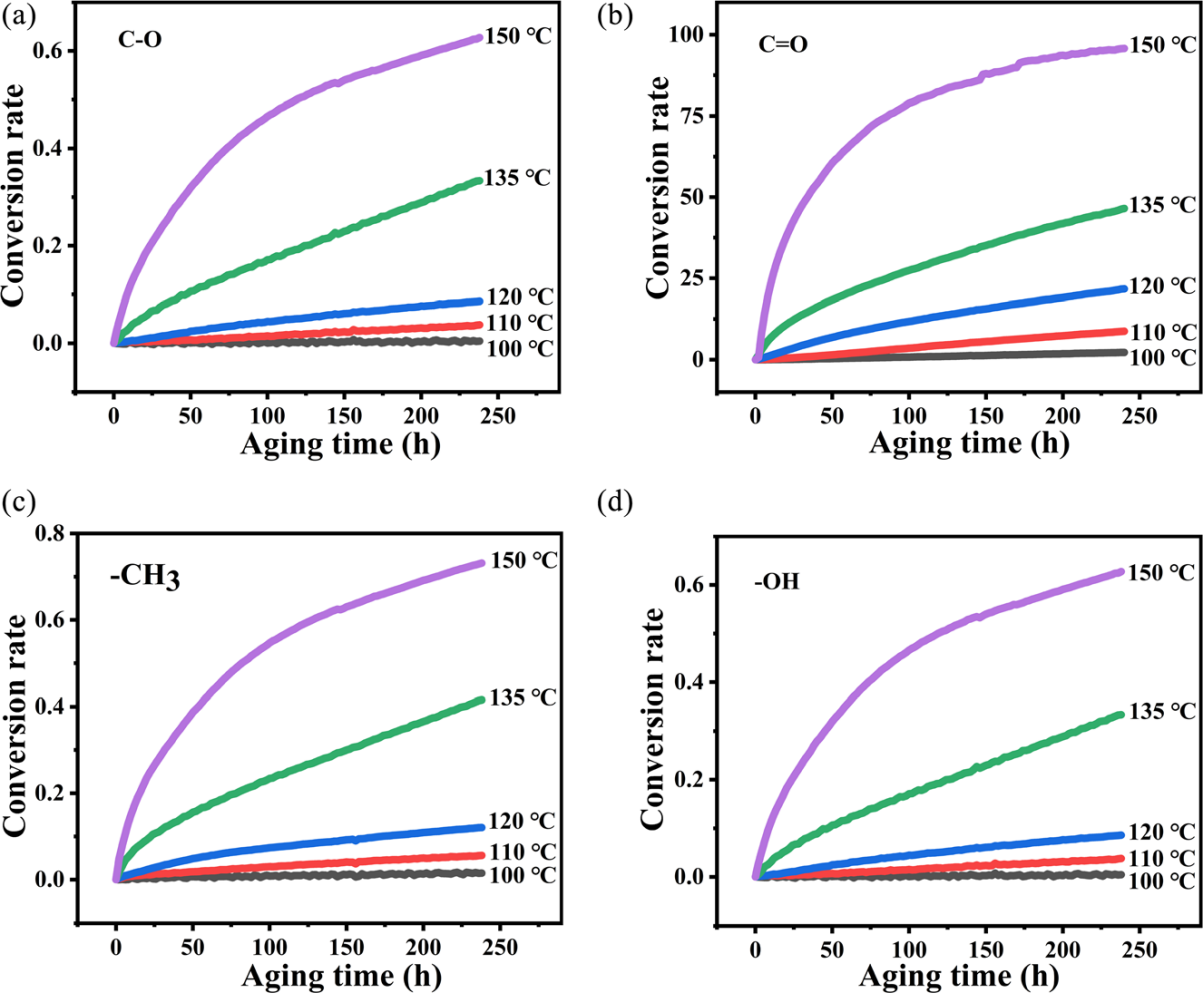
The variation of absorbance of characteristic peaks with time at different temperatures (Δ*A*–*t*): (a) C–O, (b) C=O, (c) –CH_3_, (d) –OH.

*E*_α_ and *A* are derived from the slope and intercept of the linear correlation between ln *k* and *T*^−1^ by using eqn (3). The *k* values at different vibration peaks of PVB at different temperatures can be calculated by the relationship between conversion and time, as shown in Fig. 9 and Table 2.

**Table 2:** *T* and *k* of vibration peak

Wavenumber/cm^−1^	*T*/K	*k*/s^−1^	*r*
3500	373.15	9.40 × 10^−5^	0.9152
	383.15	2.72 × 10^−4^	0.9780
	393.15	5.17 × 10^−4^	0.9675
	408.15	1.76 × 10^−3^	0.9875
2954	373.15	5.71 × 10^−5^	0.9975
	383.15	2.12 × 10^−4^	0.9907
	393.15	4.49 × 10^−4^	0.9593
	408.15	1.50 × 10^−3^	0.9837
1718	373.15	9.67 × 10^−3^	0.9976
	383.15	3.83 × 10^−2^	0.9987
	393.15	8.65 × 10^−2^	0.9815
	408.15	1.69 × 10^−1^	0.9726
1120	373.15	2.09 × 10^−5^	0.9612
	383.15	1.57 × 10^−4^	0.9940
	393.15	3.57 × 10^−4^	0.9887
	408.15	1.29 × 10^−3^	0.9925

**Fig. 10:**
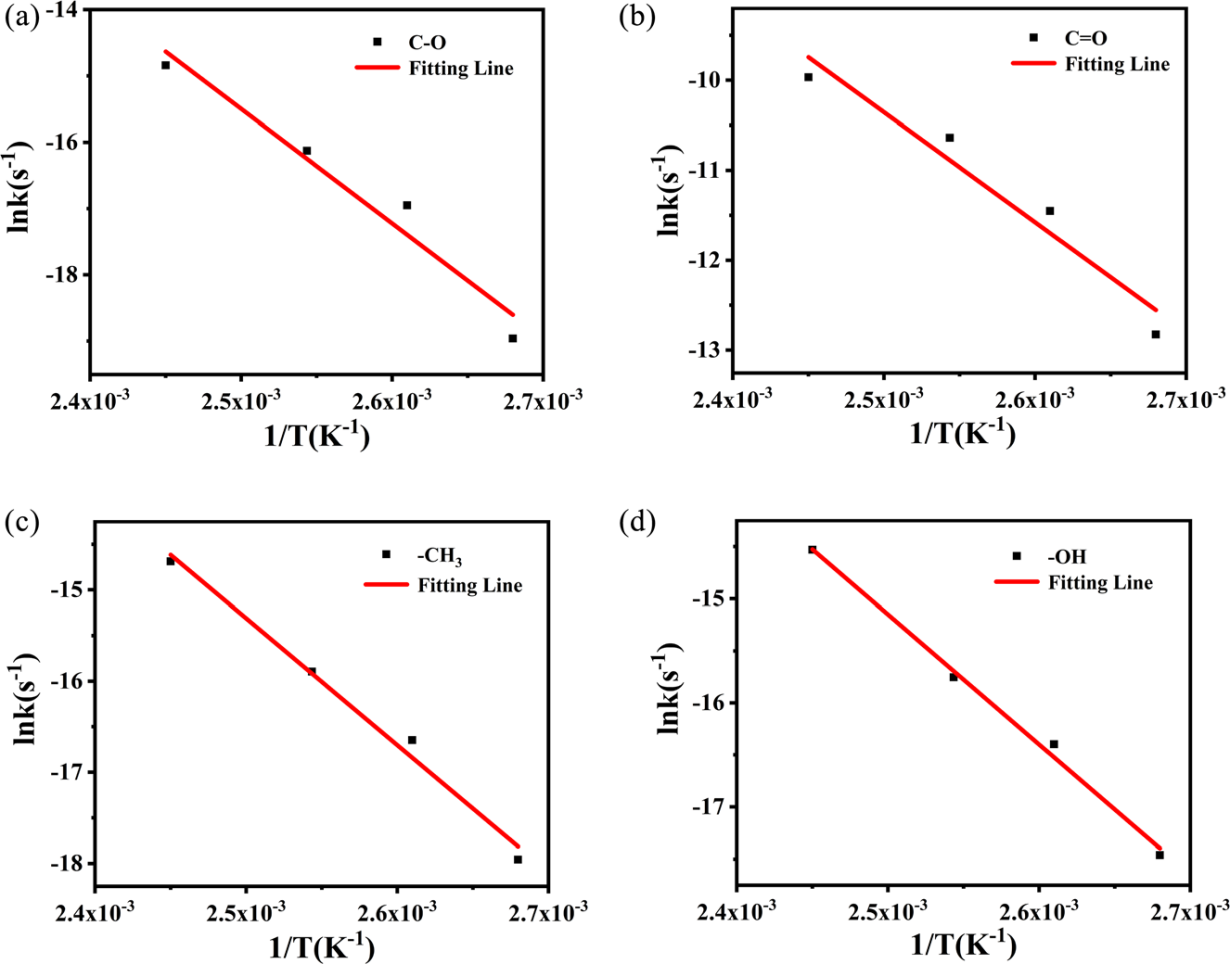
Logarithmic relationship between rate constant (ln *k*) and temperature (*T*^−1^) of methyl characteristic peak.

The values from Table 2 can be incorporated into eqn (3), and drawn as *T*^−1^ with ln *k*, as shown in Fig. 10.

The logarithmic relationship of the rate constants (ln *k*) to temperature (*T*^−1^) for the methyl characteristic peak was fitted, and other groups are also obtained by the same method. These linear relationships yield dynamic parameters for different vibration peaks, as shown in Table 3. For polymer materials suitable for the Arrhenius model, the typical value of activation energy during thermal aging is 0.75–1.6 eV, that is, 72–153 kJ mol^−1^, and even Janting^28^*et al.* have studied medical device polymer that the activation energy of thermal aging is 0.67 eV, 64.32 kJ mol^−1^. Liau^21^ and Ivanov^23^ used the characterization method of thermogravimetry and obtained the activation energy of about 200 kJ mol^−1^ through rapid thermal decomposition at high temperatures. The value of activation energy obtained cannot reflect the aging process of PVB under constant temperature. So the activation energy obtained by thermal aging is lower and more accurate than that obtained by thermal decomposition.

**Table 3:** Kinetic parameters of vibration peaks based on the Arrhenius equation

Wavenumber/cm^−1^	*E*_α_/kJ mol^−1^	*A*/s^−1^	*r*	Standard error (kJ mol^−1^)
3500	103.8	3.40 × 10^10^	0.99473	5
2954	115.7	1.02 × 10^12^	0.98862	8
1718	101.7	2.16 × 10^12^	0.94524	15
1120	143.6	3.84 × 10^15^	0.95705	21

According to the degradation of the above groups, the –C–O has the highest *E*_α_ (143.6 kJ mol^−1^), and has better stability under long-term constant temperature, followed by the –CH_3_. The *E*_α_ (103.8 kJ mol^−1^) of –OH is the lowest, indicating that –OH may be the weaknesses of the PVB molecular chain, and as the active point leading to the degradation of the PVB. The activation energies of –OH and C=O are similar, which suggests that they may have a potential conversion relationship.

#### 3.2.2 Thermal kinetic parameters analysis from the first-order reaction equation

**Fig. 11:**
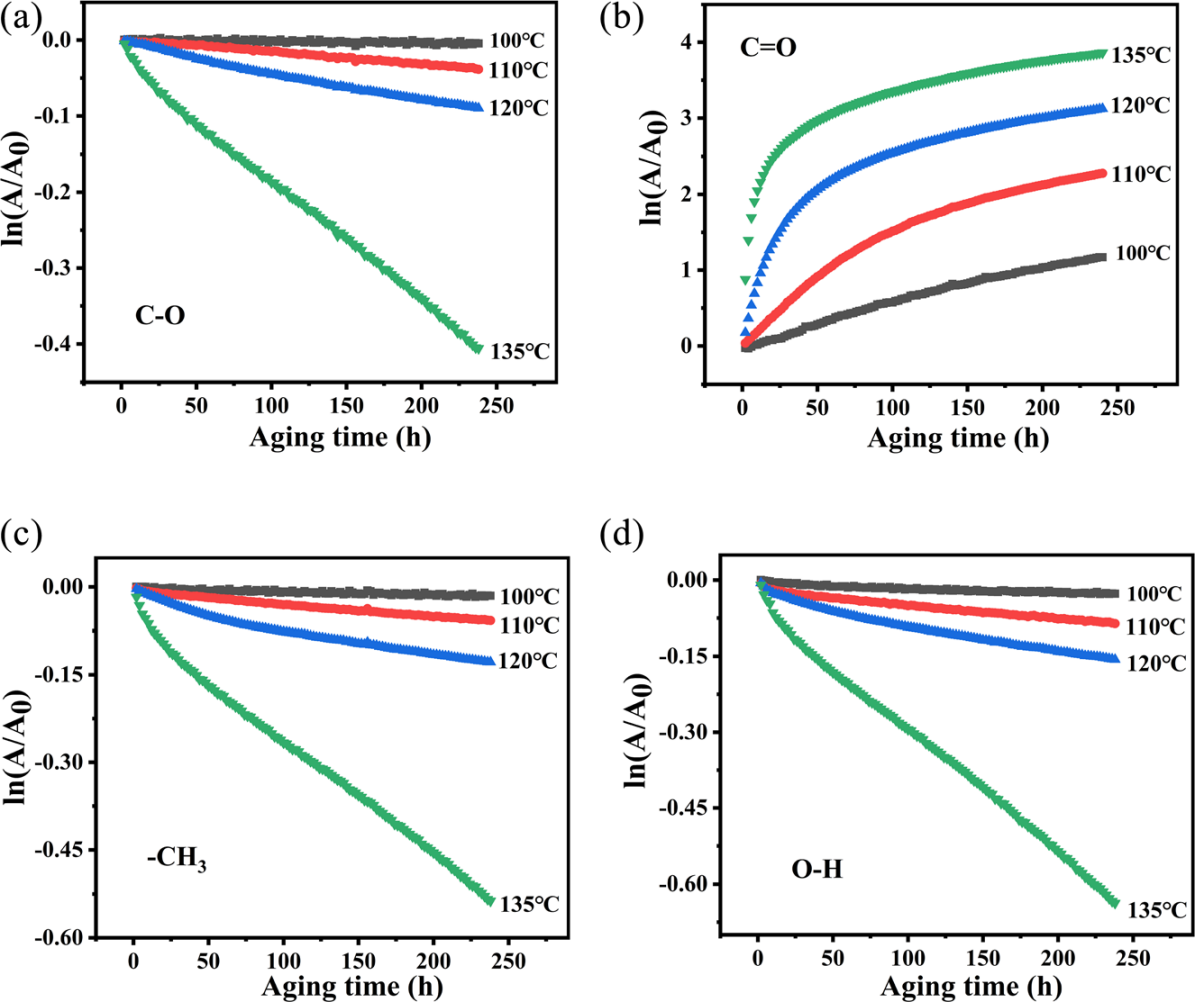
Plot of ln(*A*/*A*_0_) *versus t* of PVB at different temperatures. (a) C–O, (b) C=O, (c) –CH_3_, (d) –OH.

Since the degradation process of polymer materials conforms to the zero-order reaction or first-order reaction, we also analyzed the first-order reaction kinetics of the aging process of PVB. The first-order rate constant (*k*) was calculated using the equation, as follows:

4 ln(*A*/*A*_0_) = −*kt*

where *A*_0_ and *A* are the PVB compound absorbance at the isothermal aging time of 0 and *t*, respectively. The logarithmic dependences of (ln *A*/*A*_0_) on time (*t*) were fitted and are shown in Fig. 11. In Fig. 11, at various temperatures, the first-order reaction of the groups has a good linear relationship with time except for the carbonyl groups. Therefore, in order to study the structural changes of PVB during thermal aging, we mainly analyzed the first-order reaction model of –OH, –CH_3_ and –C–O. The *k* at different vibration peaks of PVB at different temperatures can be calculated from the relationship between first-order reaction and time, as shown in Table 4.

**Table 4:** *T* and *k* of peak vibration in first-order reaction kinetics

Wavenumber/cm^−1^	*T*/K	*k*/s^−1^	*r*
3500	373.15	9.42 × 10^−5^	0.9994
	383.15	2.82 × 10^−4^	0.9880
	393.15	5.60 × 10^−4^	0.9780
	408.15	2.44 × 10^−3^	0.9977
2954	373.15	5.71 × 10^−5^	0.9991
	383.15	2.17 × 10^−4^	0.9936
	393.15	4.78 × 10^−4^	0.9678
	408.15	1.20 × 10^−3^	0.9964
1120	373.15	2.11 × 10^−5^	0.9914
	383.15	1.60 × 10^−4^	0.9936
	393.15	3.73 × 10^−4^	0.9911
	408.15	1.58 × 10^−3^	0.9980

**Fig. 12:**
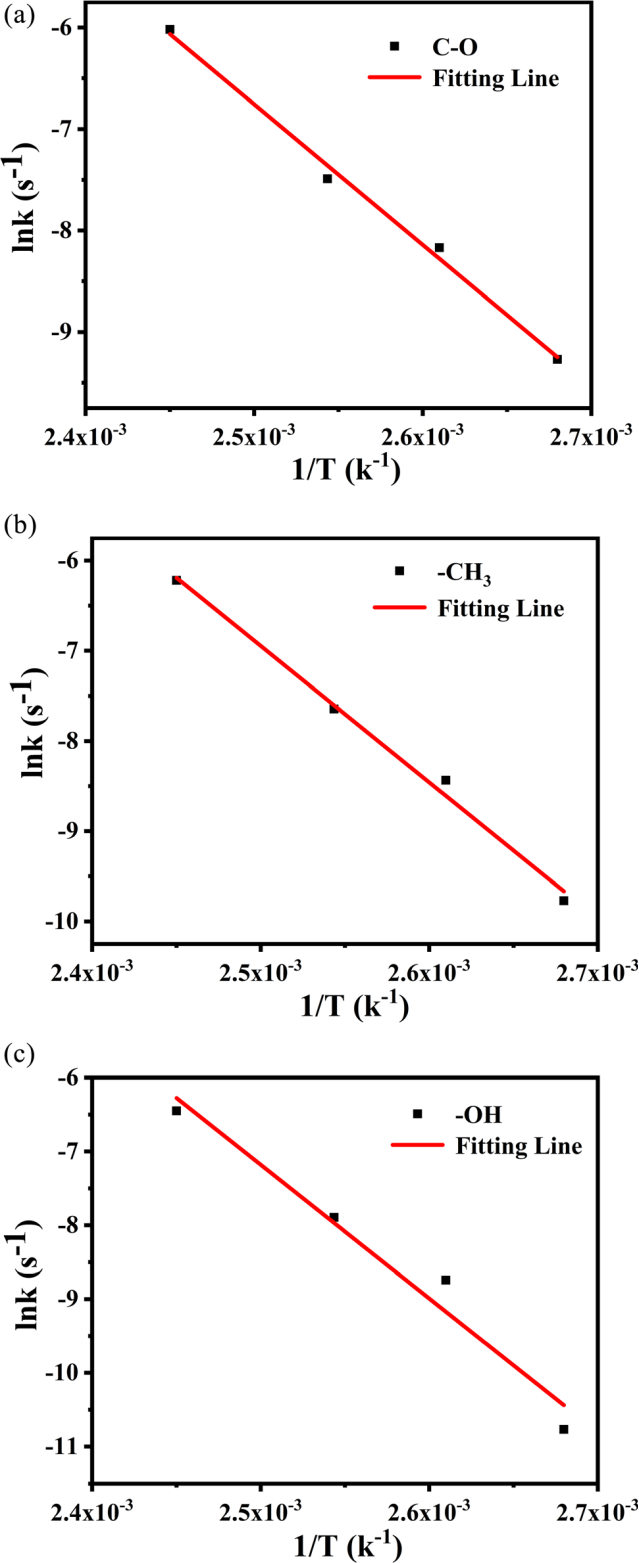
The logarithmic dependences of rate constants (ln *k*) on temperatures (*T*^−1^) of methyl characteristic peak.

The values from Table 4 can be incorporated into eqn (3) and drawn as *T*^−1^ with ln *k*, as shown in Fig. 12. The logarithmic dependences of rate constants (ln *k*) on temperatures (*T*^−1^) of methyl characteristic peak was fitted, hydroxyl and ether bond groups were also obtained by the same way. These linear relations yielded the kinetic parameters for different vibration peaks, as shown in Table 5. The *E*_α_ of –OH, –CH_3_ and –C–O are 115.2 kJ mol^−1^, 125.7 kJ mol^−1^ and 141.2 kJ mol^−1^, respectively, which suggests that –OH reacts more easily under constant temperature.

**Table 5:** Kinetic parameters of PVB based on Arrhenius equation

Wavenumber/cm^−1^	*E*_α_/kJ mol^−1^	*A*/s^−1^	*r*	Standard error (kJ mol^−1^)
3500	115.2	5.32 × 10^11^	0.9943	6
2954	125.7	2.38 × 10^13^	0.9934	7
1120	141.2	1.59 × 10^15^	0.9809	14

Based on the analysis of the above two kinetic methods, we obtained the same results. During the thermal aging process of PVB molecular chain, –C–O is relatively stable due to the highest activation energy, while the *E*_α_ of –OH is the lowest, indicating that –OH may be the weak point of PVB molecular chain and easily become the active site of PVB degradation. Secondly, based on the comparison of the linear relationships between the above two kinetics, the –OH, –CH_3_ and –C–O are more consistent with the first-order reaction kinetics, while C=O is consistent with the zero-order reaction kinetics.

### 3.3 Thermo-oxidative degradation mechanism of PVB

The thermal degradation behavior of PVB has been widely studied. The main decomposition pathway at high temperature starts from branched-chain breakage and the subsequent free radical cross-linking reaction, resulting in the loss of molecular weight of PVB and the formation and volatilization of small molecules.^21,29^ Saravanan,^30^ Liau,^22^ and Grachev^31^ also proposed the corresponding thermal degradation reaction mechanism of PVB.

According to the literature,^26,32^ the hydroxyl and acetic acid groups in PVB are greatly reduced, acetal ring groups disappear completely, and carbonyl groups increases, indicating that oxidation is one of the main factors for degradation. These conclusions are also consistent with the phenomena we have observed. And according to the conclusions of Beachell^31,32^*et al.* and Liau^21,27^*et al.*, the oxidation of carbon–hydrogen bonds and the fracture of acetal ring will form carbonyl groups. The fracture modes of acetal ring are different, and the acetal ring may form carbonyl groups after fracture. According to ref. 23, 31 and 33, PVB can be decomposed into *n*-butyraldehyde, butyric acid, acetic acid, *n*-butanol, and other substances under high-temperature thermal degradation.

Thus, based on the above conclusions, a probable mechanism for the thermal oxidative aging of PVB, which may account for these experimental observations can be summarized as follows.

**Fig. 13:**
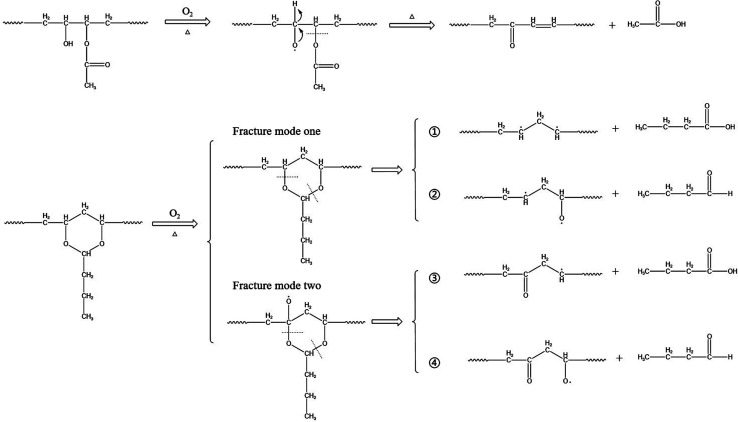
Aging mechanism of PVB.

As shown in Fig. 13 under the condition of thermal oxygen, hydroxyl dehydrogenation transfers alkoxy radicals, which are oxidized to ketones. Ester groups are also prone to breakage due to the instability of acetals and ref. 31 conclusions indicating the formation of double bonds here. According to the infrared spectrum, the absorbance of the three kinds of methyl groups decreased during thermal aging, and the C–O bond also decreased, indicating that the acetal ring was in the ring-opening process. Combining Liu^27^*et al.* and Nabil^2^^6^*et al.*, there are two main ways to break the acetal ring with high stability. One is that the C–O bonds at different positions break directly, producing two different molecular chains: ① and ②; the other way is to form oxygen free radicals by oxidation of carbon–hydrogen bonds, and then C–O bonds at different positions break, producing two kinds of molecular chains ③ and ④. Both fracture modes form free radicals, which are easily oxidized or cross-linked to form double bonds or carbonyl groups in the later stage. To further explore the decomposition mechanism of PVB, we will perform constant temperature experiments at higher temperatures through our multi-channel *in situ* reaction system.

## 4 Conclusions

The aging kinetic parameters and aging mechanism of PVB materials were investigated using multi-channel *in situ* infrared spectroscopy. The *in situ* infrared spectrum of the chemical structures of PVB materials during aging was analyzed. The *E*_α_ of –C–O, –C=O, –CH_3_, and –OH were obtained by two kinetic methods. The –OH were the weakness of PVB during its thermal aging, and the zero-order reaction shows that there is a potential conversion relationship between –OH and C=O. And then the –OH, –CH_3_ and –C–O are more consistent with the first-order reaction kinetics, while C=O is consistent with the zero-order reaction kinetics.

The above thermal aging study of PVB illustrated that the multi-channel *in situ* infrared spectroscopy could provide intuitive data on the chemical structure of materials. The combination of thermal aging kinetics and other data processing methods can deepen understanding of the thermal properties and aging process of materials. Therefore, a multi-channel device combined with *in situ* infrared spectroscopy is an effective characterization method for the material aging process.

## Conflicts of interest

There are no conflicts to declare.

## Supplementary Material
